# Homeostatic Plasticity in Epilepsy

**DOI:** 10.3389/fncel.2020.00197

**Published:** 2020-06-26

**Authors:** Gabriele Lignani, Pietro Baldelli, Vincenzo Marra

**Affiliations:** ^1^Department of Clinical and Experimental Epilepsy, Queen Square Institute of Neurology, University College London, London, United Kingdom; ^2^Department of Experimental Medicine, University of Genoa, Genoa, Italy; ^3^IRCCS Ospedale Policlinico San Martino, Genoa, Italy; ^4^Department of Neuroscience, Psychology and Behaviour, University of Leicester, Leicester, United Kingdom

**Keywords:** homeostatic plasticity, epilepsy, excitation inhibition balance, gene therapy, synaptic transmission, REST (RE-1 silencing transcription factor)

## Abstract

In the healthy brain, neuronal excitability and synaptic strength are homeostatically regulated to keep neuronal network activity within physiological boundaries. Epilepsy is characterized by episodes of highly synchronized firing across in widespread neuronal populations, due to a failure in regulation of network activity. Here we consider epilepsy as a failure of homeostatic plasticity or as a maladaptive response to perturbations in the activity. How homeostatic compensation is involved in epileptogenic processes or in the chronic phase of epilepsy, is still debated. Although several theories have been proposed, there is relatively little experimental evidence to evaluate them. In this perspective, we will discuss recent results that shed light on the potential role of homeostatic plasticity in epilepsy. First, we will present some recent insights on how homeostatic compensations are probably active before and during epileptogenesis and how their actions are temporally regulated and closely dependent on the progression of pathology. Then, we will consider the dual role of transcriptional regulation during epileptogenesis, and finally, we will underline the importance of homeostatic plasticity in the context of therapeutic interventions for epilepsy. While classic pharmacological interventions may be counteracted by the epileptic brain to maintain its potentially dysfunctional set point, novel therapeutic approaches may provide the neuronal network with the tools necessary to restore its physiological balance.

## Introduction

Epilepsy is a heterogeneous group of complex diseases, with intricate temporal profiles. In many common forms such as temporal lobe epilepsy associated with hippocampal sclerosis, the brain undergoes a process of epileptogenesis, culminating in the symptomatic, chronic phase, characterized by interictal discharges and overt seizures (Devinsky et al., 2018). It stands to reason that the cellular and molecular processes linked to the development of epilepsy would follow an equally complex temporal profile. Similarly, the brain’s intrinsic mechanisms to counter the detrimental effects caused by epilepsy are likely to be differentially regulated in epileptogenesis and the chronic epileptic phase. A simplistic interpretation of epileptogenesis is that it is a process that results in an imbalance of excitation and inhibition. However, a more complete understanding of epilepsy requires the inclusion of multiple dimensions, e.g., anatomy, synaptic and cellular features, transcriptome, and circuits dynamics. These dimensions in the phase space of the brain may have very different temporal dynamics and are, given biological constraints, often non-orthogonal. The healthy brain is a dynamic system that operates, most of the time, within certain boundaries in its physiological multidimensional zone while epileptogenic factors pull its trajectories towards pathological regions. In epilepsy, the brain crosses these boundaries more often, eventually resulting in seizures, so it can be defined as a continuous interchange between epileptic/pathological and physiological brain states associated with the occurrence of epileptic activity (Abreu et al., 2019). The physiological mechanisms that can confine the brain’s state inside healthy phase-space boundaries, despite epileptogenic attractors, fall squarely within the definition of homeostatic plasticity (Turrigiano, 2012). While many examples of homeostatic downscaling in the face of disinhibition or overexcitation can be observed *in vitro* or *ex vivo* (Grubb and Burrone, 2010; Sun and Turrigiano, 2011; Barnes et al., 2017; Xu and Pozzo-Miller, 2017; Chowdhury et al., 2018), hyperexcitability-induced homeostatic plasticity is a relatively less characterized phenomenon in complex systems *in vivo* (Lee and Kirkwood, 2019). In particular, the role of the homeostatic machinery once chronic epilepsy has been established is still unknown. In principle, in a persistently hyperexcitable network, homeostatic mechanisms should bring the brain state back to a physiological space, but this is not what has been observed in rodent models and human patients (André et al., 2018). One of the characteristic features of an epileptic brain is an aberrant recurrent hyperactivity not present in non-pathological circuits (Chang and Lowenstein, 2003). Therefore, by definition, an epileptic brain is one in which homeostatic plasticity fails to maintain the network’s physiological boundaries. Several alterations, probably alongside compensations, occur during the epileptogenesis period leading to hyperexcitable circuits which cannot be compensated by homeostatic plasticity, leaving the brain in an abnormal state and eventually causing seizures. The shift to a pathological state can be also related to the transition between interictal and ictal activity (Khambhati et al., 2017). A possibility is that pathogenic events shift the homeostatic equilibrium closer to the transition point between interictal and ictal states, effectively making the plastic changes maladaptive. While plausible, this hypothesis is difficult to test in a highly dynamical system where the homeostatic set “point” is constantly shifting in response to Hebbian and homeostatic perturbations.

### Why Is Homeostatic Plasticity Unable to Suppress Seizures in Epilepsy?

The precise mechanisms by which seizures arise are still debated, but it is widely speculated that some circuits become overactive (Devinsky et al., 2018). Why homeostatic plasticity is not able to counteract this aberrant network activity is still unknown. One possibility, plausible in acquired epilepsies, is that a gradual weakening of the homeostatic response or a maladaptive compensation may be due to the progressive neuronal degeneration during epileptogenesis. The loss of a small percentage of interneurons may have huge consequences in the network’s ability to maintain the brain in its physiological space (Houweling et al., 2005; Cossart, 2014; Queenan et al., 2018). Another possibility, that would better explain genetic epilepsies, is that the homeostatic processes occurring during the epileptogenesis, e.g., compensation of a mutated gene function, maybe at the basis of the hyperactive network observed in the chronic phase, because of the impossibility of a biological system to constantly compensate for the chronic loss of key proteins fundamental to maintain the brain within physiological boundaries.

In both cases, the failure of homeostatic plasticity in suppressing network hyperexcitability may be attributed to a failure of cellular and/or molecular mechanisms that would normally re-establish and constantly maintain the network’s physiological boundaries.

## Temporal Profile of Homeostatic Adaptations in Epilepsy

Neuronal networks are highly dynamic systems that require appropriate compensation. Homeostatic plasticity can act on a variety of different sub-cellular signaling cascades to regulate activity (Wefelmeyer et al., 2016). Similarly, the temporal profile of homeostatic plasticity must evolve to follow the network’s requirements for regulation with minimal disruption of its function. An example observed in non-pathological conditions is the developmental change in the synaptic valence of GABAergic transmission. While in the mature brain opening of GABAAR commonly leads to an influx of chloride ions and subsequent hyperpolarization, in early development chloride concentration is higher in the cell leading to GABAAR-mediated depolarization due to the efflux of chloride ions. A recent study of a specific type of interneurons, Chandelier cells, shows that, in response to sustained stimulation, GABAergic inputs are homeostatically reduced in early developmental stages (high intracellular chloride) and increased in the mature system when GABA has an inhibitory effect (Pan-Vazquez et al., 2020). This example underlines how homeostatic processes are developmentally regulated and that the direction of the compensations is dynamically guided by the network state rather than be simply fixed at the single-cell level. Experimental models of epilepsy offer a unique opportunity to study the evolution, and failures, of homeostatic processes. Most of these models introduce a perturbation in the system that leads to expanding the boundaries of the brain’s trajectory towards seizure space. In some cases, these extensions in phase space trajectory outlast the duration of the perturbation, as in the case of intracranial infusion of Tetanus toxin (TeNT). The TeNT is a small protein that impairs preferentially GABAergic release by cleaving Synaptobrevin2 at interneuron terminals (Schiavo et al., 2000). Intracranial injections of TeNT are used to induce epilepsy with two distinct phases: (I) an acute phase with a high number of ictal events and detectable TeNT activity; and (II) a chronic phase with no TeNT activity and a slowly decreasing number of ictal events (Jefferys et al., 1995; Mainardi et al., 2012; Wykes et al., 2012; Vannini et al., 2016; Chang et al., 2018; Snowball et al., 2019). In this model, the impairment of a key “homeostatic tool” prevents the system from reaching its physiological set point. However, looking at the ultrastructural level, homeostatic mechanisms are still put in place to reduce the network’s hyperexcitability. In mice injected with TeNT in the visual cortex, the active zone length of inhibitory synapses is significantly increased in the acute phase of TeNT-induced focal epilepsy (Vannini et al., 2020). At this stage. increase in active zone size is unlikely on its own to have a major effect on GABA release, given the continued catalytic effect of TeNT at this stage of the model. At a later point, changes occur in the organization of the functional fraction of excitatory vesicles. Synaptic release in response to mild visual stimulation is similar in control and epileptic mice but vesicular positioning within the presynaptic terminal is considerably different. While in control conditions release-competent vesicles are spatially biased toward the active zone (Marra et al., 2012; Rey et al., 2015), in the chronic phase of TeNT-induced epilepsy functional vesicles are evenly distributed within the cluster, presumably reducing synchronization of excitatory vesicles’ release (Vannini et al., 2020). It is plausible that increasing the average distance between functional vesicles and release site has an impact on temporal and filtering properties of excitatory synapses, changing the synaptic transfer function (action potential to vesicular release) so that high-frequency firing (typical of seizure) has a lower output while leaving information transmission within healthy space relatively unchanged (Trigo et al., 2012; Pulido et al., 2015; Pulido and Marty, 2017; Miki et al., 2018). The change in the positioning of release-competent vesicles is preceded by a sustained increase in Carboxypeptidase E, a protein required for vesicle positioning in the proximity of their release site (Park et al., 2008; Lou et al., 2010; Vannini et al., 2020). This adaptation of homeostatic mechanisms over time, and across different neuronal types, is an example of the complex and dynamic processes involved in maintaining neuronal function within healthy boundaries by acting on seemingly independent synaptic features. However, if the time course of the homeostatic response does not match closely the one of its triggering cause, plasticity may lead to a maladaptive regulation of network activity. For example, ischemic events or traumas may transiently impair neurotransmission and as a result, the system will compensate for reduced excitatory inputs becoming less stable and more likely leading to an ictal state.

## RE-1 Silencing Transcription Factor’s (Rest’s) Janus Role in Homeostatic Plasticity/Epileptogenesis

The temporal dynamic adaptation of homeostatic processes during the epileptic phases is also reflected at the transcriptomic level by differential changes in the regulation of gene expression. Indeed, growing evidence demonstrates that the same homeostatic transcriptomic pathways which in some conditions favor the recovery of a physiological set point, in other conditions exert the opposite action exacerbating neuronal hyperactivity (Baldelli and Meldolesi, 2015).

A clear example is offered by the debated role of RE-1 Silencing Transcription Factor (REST), also known as neuron-specific silencing factor (NRSF), in homeostatic plasticity. This gene-silencing transcription factor, widely expressed during embryogenesis, exerts a strategic role (Ballas et al., 2001; Roopra et al., 2001; Ooi and Wood, 2007) during the late stages of neuronal differentiation when the loss of REST is critical for the acquisition of the neuronal phenotype (Su et al., 2006).

In mature neurons, REST exhibits several unique properties. Indeed, its expression is increased by kainate-induced seizures *in vivo* (Palm et al., 1998; Gillies et al., 2009) and chronic hyperactivity in cultured neuronal cultures (Pozzi et al., 2013). Interestingly, REST induces firing homeostasis by downregulating voltage-gated Na^+^ channel expression in excitatory neurons (Pozzi et al., 2013) and scales down the strength of excitatory synapses, acting presynaptically, in response to chronic hyperactivity (Pecoraro-Bisogni et al., 2018). Because REST knockdown impairs both intrinsic and synaptic homeostasis, these results indicate that REST function is critical for inducing homeostatic negative feedback responses to readjust the network firing activity at a physiological set point and protect it from hyperactivity. Following this homeostatic role, a 2-deoxy-D-glucose ketogenic diet was reported to have an antiepileptic effect *via* the activation of a chromatin remodeling complex controlled by an increase in REST (Garriga-Canut et al., 2006) and, in the kindling model of epileptogenesis, conditional REST deletion in excitatory neurons of the postnatal mouse forebrain resulted in a dramatic acceleration of seizure progression and prolonged after-discharge duration compared with control mice (Hu et al., 2011).

On the other hand, in the kainate mouse model of temporal lobe epilepsy, blocking REST function repressed the expression of the hyperpolarization-activated, cyclic nucleotide-gated channel (HCN1) attenuating the epileptic phenotype (McClelland et al., 2011). Subsequently, the same authors revealed that the repression resulting from REST increase was not limited to HCN1 but also included 10% of the analyzed target genes. REST inhibition was found to lead to attenuation of seizures, strongly supporting the hypothesis that seizure-induced increases of REST contribute to epileptogenesis *via* REST-mediated repression of a group of genes that critically influence neuronal function (McClelland et al., 2014).

These contrasting effects still prevent us from concluding whether inhibition or enhancement of REST signaling should prevent epileptogenesis. To address this question, it will be essential to evaluate how REST changes its influences depending on conditions, for example, cell specificity, neural networks, expression timing and loci, and status of progression of epilepsy. However, a reading key that could permit to better interpret why REST-signaling is in some cases homeostatic while exerts in other cases an opposite pro-epileptogenic action, is to consider that probably the primary tasks of homeostatic plasticity is aimed to constitute a constrain at the saturation of use-dependent Hebbian plasticity (Turrigiano, 2012; Li et al., 2019).

Therefore, the homeostatic efficacy of REST is possibly effective in the initial stages of epileptogenesis, when the level of hyperactivity has not yet turned away the neuronal network too far from its physiological space. Indeed, considering that REST-signaling is strictly dependent on the neuronal hyperactivity, when this assumes excessive values, due to chronic pathological conditions, the complete dysregulation of the REST-pathway could transform its homeostatic capacity in a pro-epileptogenic function, thus contributing to the consolidation and aggravation of chronic epilepsy.

Nevertheless, homeostatic plasticity can still be considered as a therapeutic target for the protection from epilepsy even in chronic epileptic conditions, thanks to the possibility of subtracting homeostatic plasticity from the control of advanced pathological hyperactivity, through its direct and exogenous modulation by pharmacological and genetic strategies.

## The Complex Unique Properties of the Hyperpolarization-Activated Cation Current (Ih) in Homeostatic Plasticity and Epilepsy

In many cases, the same molecular actors playing crucial roles in homeostatic plasticity are, in a different time or place, fundamental mediators of the epileptogenic processes. A paradigmatic example of such complex interpenetration between homeostatic plasticity and epileptogenesis is offered by the Ih, that in recent years was ascribed as a central player of both homeostatic and epileptogenic processes. Ih is mixed sodium and potassium conductance generated by the hyperpolarization-activated cyclic nucleotide-gated (HCN) channels and activated by membrane hyperpolarization. Initially discovered in the pacemaker heart sinoatrial node cells and subsequently found to be widely expressed in the central and peripheral nervous system (Brown et al., 1979; Robinson and Siegelbaum, 2003). Ih plays an important role in determining membrane potential and firing characteristics of neurons and therefore is a potential target for homeostatic regulation. Indeed, in CA1 pyramidal cells, Ih was found to be up- or down-regulated following chronic (48 h) hyperactivity or activity deprivation, respectively. Such bidirectional homeostatic regulation not only controls spiking activity but also stabilizes the threshold for long-term potentiation induced in CA1 pyramidal neurons by repetitive stimulation, accelerating EPSP kinetics, and reducing temporal summation of EPSPs (Gasselin et al., 2015). These results suggest that modulation of Ih represents a homeostatic plasticity mechanism, allowing neurons to control their excitability and EPSP summation in response to changes in synaptic activity on both short and long-term time scale. Furthermore, the earliest reports of HCN channel dysfunction in epilepsy revealed the enhancement of somatic Ih in hippocampal CA1 pyramidal neurons of animal models of febrile seizures, and more recently, in a mouse model of fragile X with audiogenic seizures (Chen et al., 2001; Bender et al., 2003; Dyhrfjeld-Johnsen et al., 2008; Brager et al., 2012). These results were unexpected, as an increase in Ih is considered to be inhibitory, questioning that these changes may be epileptogenic and suggesting that they can potentially reflect a homeostatic process in response to augmented neural network activity.

However, in contrast with the above-mentioned results, in most experimental paradigms for investigation of recurrent epileptic seizures following administration of convulsant agents, a reduction of Ih was observed in multiple cortical and hippocampal regions (Shah et al., 2004; Jung et al., 2007; Marcelin et al., 2009). Furthermore, the deletion of HCN1 in mice resulted in greater seizure susceptibility (Huang et al., 2009; Santoro et al., 2010) and loss of function mutations in HCN1 have been recently reported causing severe neonatal epileptic encephalopathies (Marini et al., 2018).

In summary, these data can be explained only considering the complex and dynamic role exerted by the modulation of HCN channels in both epilepsy and homeostatic plasticity. The timing is crucial, with different regulation of HCN1 in the epileptic phases, with a decrease in expression during epileptogenesis followed by an increase, potentially homeostatic, in the chronic phase. On the other hand, HCN1 localization, indirect action, and overall transcriptome could influence its function. A decrease in HCN channels expression will hyperpolarize the resting membrane potential (RMP) inhibiting neuronal excitability, but at the same time, it will increase the membrane input resistance (R_in_), exerting an excitatory action, because of the reduction of the amount of current needed to depolarize the cell (Kase and Imoto, 2012). Moreover, HCN channels are differentially expressed across the brain and neuronal populations, showing also a heterogeneous subcellular distribution, with high expression in the dendrite and lower expression in the soma (Magee, 1999). Importantly Ih net effect on excitability depends on the cell-specific interplay of passive and active membrane conductance. Indeed, multiple reports have shown that Ih currents affect particularly the activity of other co-expressed subthreshold conductances (George et al., 2009; Amarillo et al., 2014; Hu and Bean, 2018). The final effect of HCN modulation therefore will depend on the specific combination of such subthreshold conductances that change in different neuronal populations and consequently the net outcome of a similar Ih modulation can be a reduced excitability in some cases and increased excitability in others.

The HCN example highlights how the role of homeostatic plasticity in epilepsy is complex and dynamic, depending not simply on temporal and spatial expression of a gene and its protein, but also the interactions between differently expressed proteins and thus on the overall transcriptome and proteasome.

## Therapeutic Interventions Based on the “Genetic Lead” of Homeostatic Compensations

Gene therapy for epilepsy is a promising approach to treat the chronic phase of the pathology (Kullmann et al., 2014). Recent gene therapies target the symptoms (seizures) rather than the cause of epilepsy, for example, decreasing the excitability of excitatory neurons or potentiating inhibitory tone (Richichi et al., 2004; Noè et al., 2008; Wykes et al., 2012; Krook-Magnuson et al., 2013; Kätzel et al., 2014; Lieb et al., 2018; Agostinho et al., 2019; Wickham et al., 2019; Colasante et al., 2020). These therapies have been efficient in decreasing intrinsic neuronal excitability, synaptic transmission, and the number of seizures, in rescuing cognitive defects and also in resetting a physiological transcriptomic profile. In these cases, no homeostatic compensations have been observed to counteract the decreased excitability induced by the therapeutic approach. Furthermore, a net positive effect at transcriptomic level induced by an increase of endogenous Kv1.1 using CRISPRa, suggests a compensatory mechanism in line with a response to an increased network activity (Colasante et al., 2020). This effect was surprising because of the uncertainty on the effect of gene therapy: does it only increase seizure threshold or does it also rescue the epileptogenic process pushing back the brain state within its physiological boundaries? Showing cognitive deficits and gene expression rescue, the data pointed towards the latter. Importantly, Kv1.1 has been recently associated with *in vivo* homeostatic response, where to compensate network hyperexcitability, neurons increased endogenous Kv1.1 expression leading to a clear reduction of their firing rate (Morgan et al., 2019). These data together provide new insights, but also open a new series of questions, on the possible homeostatic mechanisms occurring in chronic established epilepsy. It indicates that the brain is in a chronic altered state in which homeostatic plasticity cannot maintain activity in its physiological boundaries, but that with a “homeostatic boost” in the direction of a reduction in neuronal excitability, the entire network may be able to rearrange itself to a less excitable state and take back control of phase-space regulation. Because Kv1.1 is implicated in fast homeostatic response *in vivo*, its increase could potentially drive the network compensation observed (Morgan et al., 2019). Indeed, the potential homeostatic role of Kv1.1 expression has been also corroborated in other experimental settings. It has been shown that Kv1.1 reduction improves spike timing precision and thus synchronization, therefore an increase in Kv1.1 expression could desynchronize the hypersynchronous epileptic network and in this way decrease network activity (Cudmore et al., 2010). An example is an increase of Kv1.1 in Dentate Gyrus in a mouse model of TLE as a result of positive compensations to delay AP and decrease neuronal excitability (Kirchheim et al., 2013). Furthermore, Kv1.1 expression is tightly correlated with the expression of other potassium channels, such as Kv7. Indeed, a homeostatic switch from Kv1.1 to Kv7.2 in the AIS after input deprivation in the avian cochlear nucleus has been shown to increase neuronal excitability, and on the other hand, hyperexcitation induced by Kv7 inhibition results in a fast intrinsic homeostatic response in line with a possible Kv1.1 increase in the AIS (Kuba et al., 2015; Lezmy et al., 2020). These data provide important pieces of evidence of the pivotal role of Kv1.1 in the homeostatic process and how its enhancement could lead to a remodeling of the pathological hyperexcitable network in epilepsy.

However is also possible that the Kv1.1 enhancement just increased the threshold for seizure generation (unlikely because not observed in acute seizure induction), that in turns allow a rearrangement of network activity with a consequent resetting of the transcriptional profile and physiological brain state (Colasante et al., 2020). Another possible explanation is that decreasing neuronal excitability to a certain extent, the system can be pushed back to a more stable interictal state, less likely to fluctuate go back into the ictal state. This hypothesis needs to be tested experimentally with depth electrode recordings in the epileptic focus, to capture the interictal activity before and after a gene therapy treatment.

Therefore, further experiments need to be performed to understand more in-depth this phenomenon and most importantly the duration of these compensatory effects. Furthermore, regarding genetic epilepsies, gene therapy interventions at a later stage could shed light on the rules underlying circuit rearrangement during epileptogenesis (Wykes and Lignani, 2018).

Finally, these new observations also suggest that the epileptic network is not reset at a different firing rate level but is in an unstable pathological space in which network compensation can still occur if driven by external interventions. This phenomenon underlies the importance of developing potential treatments for epilepsy based on the “genetic load” of the homeostatic plasticity mechanisms.

## Conclusion

The role of homeostatic plasticity in Epilepsy is still not fully understood, however, new insights underline its importance in the temporal and dynamic dysregulation of the neuronal network in the consolidation of this pathology. Probably many unseen homeostatic compensations occur to protect the network for being hyperactive and prevent epileptogenesis and seizures. These physiological protective processes are difficult to observe experimentally and a full understanding of the molecular mechanisms underlying seizure-induced homeostasis without the confounding of epileptogenic processes is required. Relatively simple *in vitro* systems (e.g., dissociated or slice cultures), where homeostatic plasticity works in relative isolation, maybe better experimental models for the dissection of different homeostatic mechanisms. However, each finding *in vitro* will need to be validated in the higher dimensional phase space offered by *in vivo* in models of chronic epilepsy that preferably avoids neuronal injury and death, as the discrimination between injury-induced changes and homeostatic-induced mechanisms will be critical.

**Figure 1:**
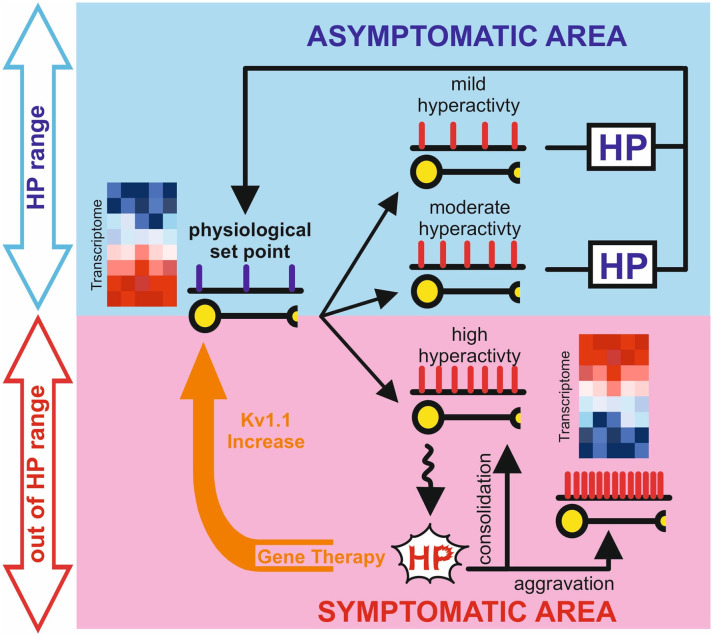
Graphical representation of the potential role of homeostatic plasticity in the epileptic process. The blue box represents the physiological space (Asymptomatic Area) in which homeostatic plasticity, operating within the limits of physiological condition, is still able to counteract changes in neuronal activity to reset the correct setpoint. The light red box represents the pathological space (Symptomatic Area), in which homeostatic plasticity has failed or even aggravate the network state. Gene therapy interventions (orange arrow) can reset the physiological set point in the chronic phase of epilepsy probably “boosting” the inefficient homeostatic plasticity. Representative heatmaps represent gene expression in physiological and pathological conditions, where blue is gene downregulation and red is gene upregulation.

Given the wide variability of expression profiles that can lead to phenotypically undistinguishable physiological outputs (Marder and Goaillard, 2006), it is likely that slightly different epileptogenic histories may lead to engaging different and seemingly independent, homeostatic mechanisms. When the system’s state crosses the limit within which homeostatic plasticity operates (Figure 1), these compensations cannot counteract neuronal hyperexcitability leading to a maladaptive or partial compensation. Therefore, further experiments need to be performed to clarify the role of homeostatic plasticity in epilepsy. These studies are likely to require modern experimental tools to dissect the fine physiological adaptation able to prevent seizure onset as well as define their operational boundaries.

Effective gene therapies that not only decrease seizures but also rescue gene expression need to be studied in more detail to understand the cascade of events that eventually lead to restoring a physiological network. A deeper understanding of successful homeostatic compensation of pathological brain state may lead to new targets for efficient therapeutic interventions, able to work in concert with the brain’s physiological regulation of neural activity.

## Data Availability Statement

The original contributions presented in the study are included in the article, further inquiries can be directed to the corresponding author/s.

## Author Contributions

All authors contributed equally to the manuscript.

## Conflict of Interest

The authors declare that the research was conducted in the absence of any commercial or financial relationships that could be construed as a potential conflict of interest.
